# Serum protein profiling using an aptamer array predicts clinical outcomes of stage IIA colon cancer: A leave-one-out crossvalidation

**DOI:** 10.18632/oncotarget.7488

**Published:** 2016-02-19

**Authors:** Jung Wook Huh, Sung Chun Kim, Insuk Sohn, Sin-Ho Jung, Hee Cheol Kim

**Affiliations:** ^1^ Department of Surgery, Samsung Medical Center, Sungkyunkwan University School of Medicine, Seoul, Korea; ^2^ Biois Corp, Seoul, Korea; ^3^ Biostatistics and Clinical Epidemiology Center, Samsung Medical Center, Seoul, Korea; ^4^ Department of Biostatistics and Bioinformatics, Duke University, Durham, NC, USA

**Keywords:** aptamer, colon cancer, prognosis

## Abstract

**Background:**

In this study, we established and validated a model for predicting prognosis of stage IIA colon cancer patients based on expression profiles of aptamers in serum.

**Methods:**

Bloods samples were collected from 227 consecutive patients with pathologic T3N0M0 (stage IIA) colon cancer. We incubated 1,149 serum molecule-binding aptamer pools of clinical significance with serum from patients to obtain aptamers bound to serum molecules, which were then amplified and marked. Oligonucleotide arrays were constructed with the base sequences of the 1,149 aptamers, and the marked products identified above were reacted with one another to produce profiles of the aptamers bound to serum molecules. These profiles were organized into low- and high-risk groups of colon cancer patients based on clinical information for the serum samples. Cox proportional hazards model and leave-one-out cross-validation (LOOCV) were used to evaluate predictive performance.

**Results:**

During a median follow-up period of 5 years, 29 of the 227 patients (11.9%) experienced recurrence. There were 212 patients (93.4%) in the low-risk group and 15 patients (6.6%) in the high-risk group in our aptamer prognosis model. Postoperative recurrence significantly correlated with age and aptamer risk stratification (p = 0.046 and p = 0.001, respectively). In multivariate analysis, aptamer risk stratification (p < 0.001) was an independent predictor of recurrence. Disease-free survival curves calculated according to aptamer risk level predicted through a LOOCV procedure and age showed significant differences (p < 0.001 from permutations).

**Conclusion:**

Aptamer risk stratification can be a valuable prognostic factor in stage II colon cancer patients.

## INTRODUCTION

Colon cancer is the second most common cause of cancer-related death in Western society and Asian countries, and approximately 40% of patients have lesions that are classified as stage II. [1] High-risk factors such as T4 lesions, poorly differentiated cell type, lymphovascular invasion, and perineural invasion have been proposed as indicators of the need for adjuvant chemotherapy; however, the role of chemotherapy in patients without these risk factors (stage IIA) is still controversial and unclear. [2] The depth of tumor invasion into the bowel wall and lymph node metastasis are the strongest predictors of colon cancer recurrence; however, colon cancer lesions of the same stage may have different clinical courses and oncologic outcomes. The stage alone cannot accurately describe the clinical behaviors of colon cancer. There is evidence that biomarker evaluation directly assists in improvements in colon cancer patient care by more accurately refining prognosis and contributing to the selection of the most appropriate adjuvant therapy. [3] Determination of novel tissue-based prognostic markers at the molecular level is important in these evaluations.

Aptamers are nucleic acid ligands identified from a random nucleic acid library as being able to bind to a wide variety of specific molecules such as proteins with high affinity and specificity using an *in vitro* iterative selection technique, called systemic evolution of ligands by exponential enrichment (SELEX). [4] Aptamers are emerging as useful agents for diagnostic and therapeutic applications, however, there are no existing reports in the field of colon cancer. Therefore, the aim of this study was to establish and validate a model for predicting prognosis in stage IIA colon cancer patients based on expression profiles of aptamers in serum.

## RESULTS

### Patient characteristics

This study included 142 men (62.6%) with a median age of 60 years (range, 29–84 years). During the median follow-up period of 5 years, 29 of the 227 patients (11.9%) experienced recurrence. The clinical and pathological characteristics in the recurred versus non-recurred groups are summarized in Table 1. Postoperative recurrence was significantly correlated with age and aptamer risk stratification (p = 0.046 and p = 0.001, respectively); however, no associations were observed between recurrence and sex, lymphovascular invasion, differentiation, the number of retrieved lymph nodes, postoperative chemotherapy, serum level of carcinoembryonic antigen (CEA), and clinical risk stratification (Table 1).

**Table 1:** Patient characteristics based on whether or not they experienced disease recurrence

	Recurrence (n = 29)	Non-recurrence (n = 198)	p-value
Age, years			0.046
<60	11 (8.9)	112 (91.1)	
≥60	18 (17.3)	86 (82.7)	
Sex			0.069
Male	14 (9.9)	128 (90.1)	
Female	15 (17.6)	70 (82.4)	
Differentiation			0.334
Well	26 (13.5)	167 (86.5)	
Moderately	3 (8.8)	31 (91.2)	
Lymphovascular invasion			0.303
Negative	24 (12.1)	174 (87.9)	
Positive	5 (17.2)	24 (82.8)	
No. of lymph nodes retrieved			0.311
<12	8 (15.7)	43 (84.3)	
≥12	21 (11.9)	155 (88.1)	
Postoperative chemotherapy			0.077
No	7 (22.6)	24 (77.4)	
Yes	22 (11.2)	174 (88.8)	
CEA*			0.550
>5	157(89.2)	19(10.8)	
≥5	28(84.8)	5(15.2)	
Clinical risk			0.475
No	17 (12.3)	121 (87.7)	
Yes	12 (13.5)	77 (86.5)	
Aptamer risk			0.001
Low	22 (10.4)	190 (89.6)	
High	7 (46.7)	8 (53.3)	

* Excluding 18 patients who were unavailable. CEA, carcinoembryonic antigen.

### Modeling categorization of expression signatures

We calculated the p-value for each aptamer using a univariate Cox regression model on 227 samples and selected 53 aptamers with p-values < 0.01 to develop a prediction interval. We used a gradient lasso algorithm to fit a prediction model. Based on variable selection, the fitted prediction model included 3 aptamers as covariates, which are listed in Table 2, together with the univariate p-values. For each of the 3 aptamers selected in the prediction model from the total data set of 227 samples, the number of times they were included in the 227 prediction models during LOOCV procedures is shown in Table 2.

**Table 2:** List of the 3 proteins included in the prediction model fitted by the total data set (n=227), their univariate p-values, and the number of models that included each protein during LOOCV

APTAMER ID	p-value	Frequency
626	<0.001	227
741	<0.001	227
42	<0.001	188

LOOCV, leave one out cross validation.

### Prognostic value of aptamer expression profiling

**Figure 3:**
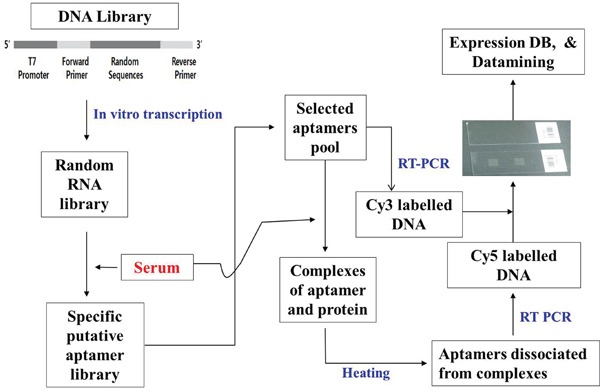
–log10 (log-rank p-value) for all possible cutoff points The dotted horizontal line denotes alpha=0.05. To divide the patients into low- and high-risk groups for DFS, the optimal cut-off was chosen as the point with the most significant log-rank p-value for all possible cut-off points.

**Figure 4:**
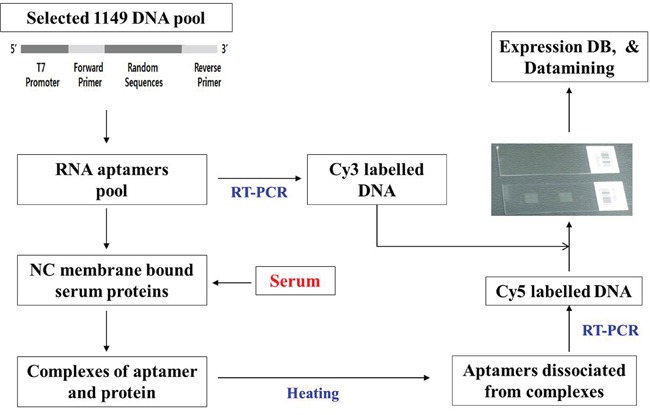
Kaplan-Meier curves for low- and high-risk groups as determined by leave-one-out cross-validation The p-value was calculated from 500 permutations (p < 0.001).

We used our prognostic model to divide the patients into low- and high-risk groups based on optimal cut-off (Table 3). The optimal cut-off is chosen as the point with the most significant log-rank p-value for all possible cut-off points (Figure 3). There were 212 patients (93.4%) in the low-risk group and 15 patients (6.6%) in the high-risk group based on the aptamer prognostic model. The aptamer risk stratification was significantly associated with postoperative recurrence; however, no associations were observed between aptamer risk stratification and age, sex, lymphovascular invasion, differentiation, the number of retrieved lymph nodes, postoperative chemotherapy, serum level of CEA, or clinical risk stratification (Table 3). Figure 3 shows the Kaplan-Meier curves for the low- and high-risk groups classified by the LOOCV procedure. The 5-year disease-free survival rates for the low- and high-risk aptamer groups were 89.8% and 53.0%, respectively (p < 0.001, Figure 4).

**Table 3:** Correlations between aptamer risk stratification and clinicopathological factors

	Low risk (n = 212)	High risk (n = 15)	p-value
Age, years			0.842
<60	114 (53.8)	9 (60.0)	
≥60	98 (46.2)	6 (40.0)	
Sex			0.949
Male	133(62.7)	9 (60.0)	
Female	79 (37.3)	6(40.0)	
Differentiation			1.000
Well	180 (84.9)	13 (86.7)	
Moderately	32 (15.1)	2 (13.3)	
Lymphovascular invasion			0.227
Negative	183 (86.3)	15 (100.0)	
Positive	29 (13.7)	0 (0.0)	
No. of lymph nodes retrieved			1.000
<12	48 (22.6)	3 (20.0)	
≥12	164 (77.4)	12 (80.0)	
Postoperative chemotherapy			0.238
No	28 (13.2)	4 (26.7)	
Yes	184 (86.8)	11 (73.3)	
CEA*			0.697
>5	164(93.2)	12(6.8)	
≥5	32(97.0)	1(3.0)	
Clinical risk			0.450
No	127 (59.9)	11 (73.3)	
Yes	85 (40.1)	4 (26.7)	
Recurrence			0.001
No	190 (89.6)	8 (53.3)	
Yes	22 (10.4)	7 (46.7)	

* Excluding 18 patients who were unavailable. CEA, carcinoembryonic antigen.

### Multivariate analysis for recurrence

We conducted a multivariate analysis to determine whether classification as low- versus high-risk from the LOOCV was an independent prognostic maker after adjustment for covariates including age, sex, postoperative chemotherapy, serum level of CEA, and clinical risk factors (Table 4). We found that aptamer risk stratification (p < 0.001) was an independent predictor of recurrence.

**Table 4:** Multivariate Cox regression analysis for recurrence according to the risk level determined by LOOCV

	Hazard Ratio (CI)	p-value
Age	2.11 (0.90–4.94)	0.084
Sex	1.95 (0.87–4.37)	0.105
Postoperative chemotherapy	0.79 (0.27–2.30)	0.670
Clinical risk	1.22 (0.53–2.82)	0.640
Aptamer risk	6.78 (2.45–18.81)	<0.001
CEA	1.52 (0.53–4.34)	0.432

LOOCV, leave one out cross validation; CI, confidence interval.

### Risk of recurrence in patient subgroups

**Figure 5:**
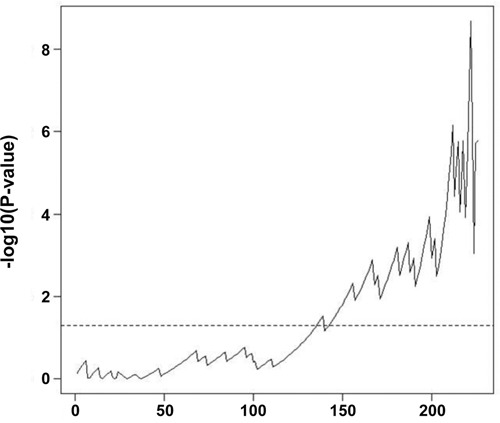
Kaplan-Meier curves for the patient groups classified by age (age < 60 years vs. age > 60 years) and risk level (low vs. high) p-value were calculated using a two-sided log-rank test (p < 0.001).

Based on the results of our multivariate analysis, we divided patients into subgroups based on age and the aptamer model (Figure 5). The 5-year disease-free survival rates of patients who were <60 years old and aptamer low-risk (n = 114), <60 years old and aptamer high-risk (n = 9), ≥60 years old and aptamer low-risk (n = 98), and ≥60 years old and aptamer high-risk (n = 6) were 93.9%, 55.6%, 85.1%, and 50.0%, respectively (p < 0.001, Figure 5) We observed that survival curves constructed according to aptamer risk level (predicted through LOOCV) and age were significantly different.

## DISCUSSION

To our knowledge, this is the first translational analysis evaluating and validating a model for prognosis prediction in stage IIA colon cancer patients based on expression profiles of aptamers in serum. The present investigation yielded 3 key results. First, we quickly produced profiles with information about patterns of the serum molecules bound to the 1,149 serum molecule-binding aptamers in a wide variety of serums samples; we then selected statistically and clinically significant aptamers. Second, the disease-free survival rates of the low- and high-risk aptamer groups were significantly different. Third, and most importantly, aptamer risk stratification was significantly associated with postoperative recurrence and was an independent predictor of recurrence in stage IIA colon cancer; moreover, this was validated using a LOOCV method.

Aptamers are short single-stranded oligonucleotides that recognize their target based on three-dimensional shape. They were identified from randomly synthesized oligonucleotide libraries following SELEX. [10] Aptamers are emerging as useful agents for diagnostic and therapeutic applications. In particular, aptamers bind soluble proteins, intracellular proteins, and cell surface antigens, which helps avoid problems associated with intracellular delivery. [11] In breast cancer, decision-making is aided by gene expression-based prognostic assays such as Onco*type* DX^®^ (Genomic Health Inc., Redwood City, CA, USA) and MammaPrint^®^ (Agendia, Amsterdam, The Netherlands), which are proven and validated for specific clinical settings. [12] These tests are already clinically available and are currently being evaluated by prospective trials. In colon cancer, however, there is little data regarding expression profiling, and the only data comes from oligonucleotide assays using tumor tissue.

We developed a technique for selection of clinically significant aptamers using complex samples containing unknown molecules. In this method, the base sequences of aptamers bound to serum molecules were determined using serum samples containing unknown molecules, allowing us to create a library. The base sequences that were complementarily bound to the selected aptamers were used to make an oligonucleotide array, and the reaction of clinically significant and well-defined serum samples with the selected serum molecule-binding aptamers was used to profile serum molecule aptamers. A database was created based on clinical information from these profiles. This technique for selection of aptamers that enables discrimination of databases via data mining is called “reverse-SELEX” (unpublished data).

Protein and aptamer arrays have been developed for high throughput screening of proteinous substances. [13, 14] We created profiles of serum molecule-binding aptamers from patient serum. Our study included 198 patients with a low-risk of colon cancer recurrence and 29 patients with a high-risk of recurrence. We used 1,149 serum molecule-binding aptamer pools that were selected by “reverse-SELEX” and an oligonucleotide array constructed using their base sequences. Our method included several stages: amplification of aptamers in the serum molecule-aptamer complexes obtained from reaction of the serum and serum molecule-binding aptamer pools using PCR and marking the products; reaction of the marked products with oligomers from the oligonucleotide array such that spots with complementary oligomers generated a fluorescent signal, which produced the serum molecule-binding aptamer profile; and finally, development of a database that included the aptamer profiles and clinical information, allowing selection of significant serum molecule-binding aptamers by statistical methods.

Patients with stage II colon cancer have a relatively good prognosis after curative surgery and the role of adjuvant chemotherapy in this subset of colon cancer patients is debatable. A small but not statistically significant improvement in oncologic outcomes was demonstrated in stage II colon cancer patients treated with adjuvant chemotherapy however, most trials included both stage II and III colon cancer, so analysis of only stage II colon cancer patients might not be available. Therefore, decisions concerning adjuvant chemotherapy must depend on subset analyses, database studies, and meta-analyses. Stage IIA colon cancer is characterized by tumor invasion that is confined within the colonic wall without nodal metastasis, and concrete recommendations concerning adjuvant chemotherapy for this group have not been incorporated into daily practice. Although there is no consensus regarding whether postoperative chemotherapy should be administrated to all patients with stage II cancer, most clinicians in Korea tend to use postoperative chemotherapy to treat these patients. [15, 16] Moreover, there is evidence that adjuvant chemotherapy may be beneficial in all patients with stage II disease. [17, 18] Pooled analysis of data from four National Surgical Adjuvant Breast and Bowel Project adjuvant studies demonstrated the benefits of adjuvant chemotherapy in stage II (Dukes’ B) patients in a manner that was not related to the presence or absence of high-risk factors. These observations support our findings for the 196 patients (86.3%) in our study who received postoperative adjuvant chemotherapy. We found that our new system using aptamers could serve as an important prognostic indicator in patients with stage II colon cancer.

In this study, we have chosen useful 3-spot profiles, i.e. identified a 3-aptamer classifier and acquired the corresponding 3 aptamers for colorectal cancer evaluation and follow-up using the 1,149-aptamer profile, which are produced by analysis of colorectal cancer samples with the 1,149-aptamer pool and 1,149-aptamer custom-made oligonucleotide microarray chip. Mammaprint, the best well known technology for *in vitro* diagnostic multivariate index assays and diagnosis of breast cancer recurrence using mRNA analysis, and diagnosis of cancer using the 70-gene classifier after analysis of total mRNA by Agilent oligonucleotide microarray chip with 1,900 features.

Based on these research results, we suggest use of the 3-aptamer classifier for evaluation of colorectal cancer and follow up using the 1,149-aptamer profile from analysis of colon cancer samples with the 1,149 aptamer pool and the 1,149 custom-made oligonucleotide microarray chip. Additionally, we suggest using only 3-aptamers selected from this research and the corresponding 3-aptamer classifier spots for evaluation and follow-up of colon cancer, however, this will require further evaluation and additional experiments.

We acknowledge the limitations of this study. The main drawback of this analysis is that our results have not been validated in an independent data set; this is of particular concern given that there are only 29 events. In addition, it appears as though the risk groups were determined based on the relationship of the aptamer panel to outcome and so it may not be surprising that there are differences in outcome between the risk groups. Moreover, we found that the disease-free survival curves in the old age groups were lower than the curves of those in the young age group in this study. Compared to the old age group, the young age group received more postoperative chemotherapy (93% vs. 78%, p-value=0.003). Although postoperative chemotherapy was not a significant prognostic factor (p=0.670), we observed that it had some positive effects on recurrence-free survival (HR=0.79, Table 4). This may potentially explain the better prognosis of the young age group within each aptamer risk group (Figure 5) that does not adjust for postoperative chemotherapy. Despite these limitations, our study has a variety of strengths. First, this is the first to evaluate the prognostic impact of the aptamer assay in stage II colon cancer. Second, our study has been validated using a specific statistical method instead an independent data set due to a small stage IIA colon cancer sample. Third, the large sample size in stage IIA patients with blood and tissue is associated with a high degree of power. Our study provides preliminary results that can help guide appropriate questions on these issues and set the stage for a validation study in the near future.

In conclusion, aptamer risk stratification can be a valuable prognostic factor in stage II colon cancer patients and it could be used as an indicator for chemotherapy.

## PATIENTS AND METHODS

Blood from 227 consecutive patients with pathologic T3N0M0 (stage IIA) colon cancer was gathered from a prospectively-collected tissue bank at Samsung Medical Center between November 2001 and February 2006. Patients with rectal cancer, synchronous tumors, and familial colon cancer were excluded from this analysis. The Institutional Review Board of Samsung Medical Center approved this study.

All patients underwent radical colonic surgery, including regional lymph node dissection. Postoperative 5-fluorouracil-based chemotherapy was recommended for all patients, regardless of clinical high-risk factors (lymphovascular invasion, poor differentiation, less than 12 lymph nodes harvested, or obstruction). The decision to administer postoperative adjuvant therapy was made after assessing the general health of the patient and with patient consent.

The patients were followed at 3-month intervals for 2 years, at 6-month intervals for the next 3 years, and then annually thereafter. Follow-up examinations were conducted on a semi-annual basis if recurrence was suspected; the examinations included clinical history, physical examination, serum carcinoembryonic antigen assay, chest radiography or computed tomography, abdominopelvic computed tomography, colonoscopy, and positron emission tomography scanning, if available. The median duration of follow-up for all 227 patients was 60.2 months (range, 15.5–99.5 months). For 29 of the 227 patients (11.9%), recurrence was confirmed by clinical and radiological examinations or by histology.

### Preparation and storage of blood samples

All blood samples were collected 1 day before surgery. Samples were centrifuged at 2,500 rpm for 15 minutes without anticoagulant. Isolated serum was deposited in 1.5 mL Eppendorf tubes and stored at −80°C.

### Aptamer platform

A randomly transformed RNA library was reacted with serum to separate nucleic acids bound to serum molecules for reverse transcriptase-polymerase chain reaction (RT-PCR). Base sequences were determined by cloning to plasmids, and a serum molecule-binding aptamer library was created. An oligonucleotide array was constructed with the base sequence of the selected aptamers. The selected serum molecule-binding aptamer library was reacted with serum, and RT-PCR was used to amplify and mark the serum molecule-binding aptamer pools. Serum molecule-binding aptamer profiles were then determined by reaction with the oligonucleotide array. For serum samples that had significant clinical information, we performed serum molecule-binding aptamer profiling and developed a database that included the serum molecule-binding aptamer profile along with the clinical information for the samples. One-way analysis of variance was used to identify spots for inclusion in the serum molecule-binding aptamer profile database: a total of 1,149 aptamers of clinical significance were identified (unpublished data). We made an oligonucleotide array with the base sequences corresponding to these 1,149 aptamers, and 1,149 serum molecule-binding aptamer pools were used in this study.

### Preparation of 1,149 serum molecule-binding aptamers

We performed PCR using DNA from the 1,149 aptamer pools to produce double-stranded DNA, followed by *in vitro* transcription of the double-stranded DNA to produce RNA aptamers. The forward primer of SEQ ID NO. 1 used in this PCR can hybridize with the 5-terminal underlined base sequence and contains a promoter base sequence for the RNA polymerase of bacteriophage T7. Alternatively, the reverse primer of SEQ ID NO. 2 used in PCR can hybridize with the 3-terminal underlined base sequence. PCR was performed using 2,500 pmoles of the PCR pair (5P7) in a buffer solution containing 50 mM KCl, 10 mM Tris-Cl (pH 8.3), 3 mM MgCl_2_, 0.5 mM dNTPs (dATP, dCTP, dGTP, and dTTP), and 0.1 U Taq DNA polymerase (Perkin-Elmer, Foster City, CA, USA) with 1,000 pmoles of single-stranded nucleic acid transcripts serving as templates. PCR products were purified using QIAquick spin columns (QIAGEN Inc., Chatsworth, CA, USA). Purified PCR product template (1 μg) was used in a standard DuraScribe T7 transcription reaction of 20 μl, where the canonical CTP and UTP were replaced with 2-F-dCTP and 2-F-dUTP, respectively. Reactions were incubated at 40°C for 4 hours. The DuraScript RNA was precipitated and purified on a spin column, and yields were determined by spectrophotometry (EPICENTRE Biotechnologies, Madison, WI, USA).

### Nucleic acid chip manufacturing

**Figure 1:**
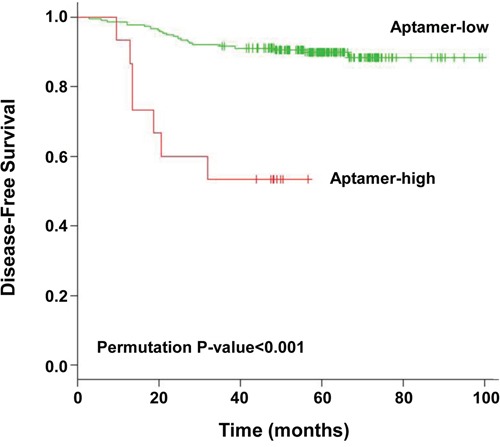
General method for the selection of 1,149 aptamers A library of engineered RNA was prepared by *in vitro* transcription of designed DNA library templates. Serum was transferred to a reaction tube after binding to nitrocellulose membrane. SELEX buffer and prepared RNA library were added to the tube for the reaction to occur. The nitrocellulose membrane was washed and heated at 95°C for 5 min, and the purified DNA was amplified by using RT-PCR. The DNA array was prepared from DNA obtained from colony PCR by using specific primers. The RNAs prepared by reaction with serum and RNA pools from previous PCR were then amplified using PCR and labeled with Cy5. Equal amounts of Cy5-labeled and Cy3-labeled products of these PCR-amplified RNA pools were allowed to react with a DNA array to generate the resulting images. A database was constructed based on the clinical information obtained from results of many different serum samples and used to analyze an array spot via one-way ANOVA. All 1,149 aptamers were selected using the methods above.

The captured single-stranded nucleic acids that we fixed on a glass slide were chemically synthesized as single-stranded nucleic acids (oligonucleotides) with base sequences that were complementary to the approximately 3,000 biomolecule-binding single-stranded RNAs determined in Figure 1 (Bioneer, Dajeon, Korea).

### Preparation of aptamer-binding serum molecules

We added 10 μL of serum to 90 μL PB. A nitrocellulose membrane disc was soaked in the mixture for 30 min with shaking. Subsequently, 100 to 400 ng of the serum molecule-binding-aptamers prepared above were added to the mixture and incubated for 30 min to form biomolecules complexes with the serum molecule-binding aptamers. After the serum sample-bound disc was treated with the prepared serum molecule-binding aptamers for 30 min, it was washed three times with a selection buffer or 50 mM ethylenediaminetetraacetic acid to remove unbound serum molecule-binding aptamers. The disc with attached complexes of serum proteins (biomolecules) with serum molecule-binding aptamers were treated RT-PCR buffer, and then RT-PCR was performed using a Cy-5 labeled primer (5-Cy5-CGGAAGCGTGCTGGGCC-3-: SEQ ID NO. 3). The serum molecule-binding aptamers prepared from the plasmid pool were subjected to RT-PCR in the same manner using a Cy-3 labeled primer (5-Cy3-CGGAAGCGTGCTGGGCC-3-). The two resulting solutions were mixed in equal volumes to target single-stranded nucleic acids.

### Reaction of the aptamer array with the serum molecule-binding aptamers

**Figure 2:**
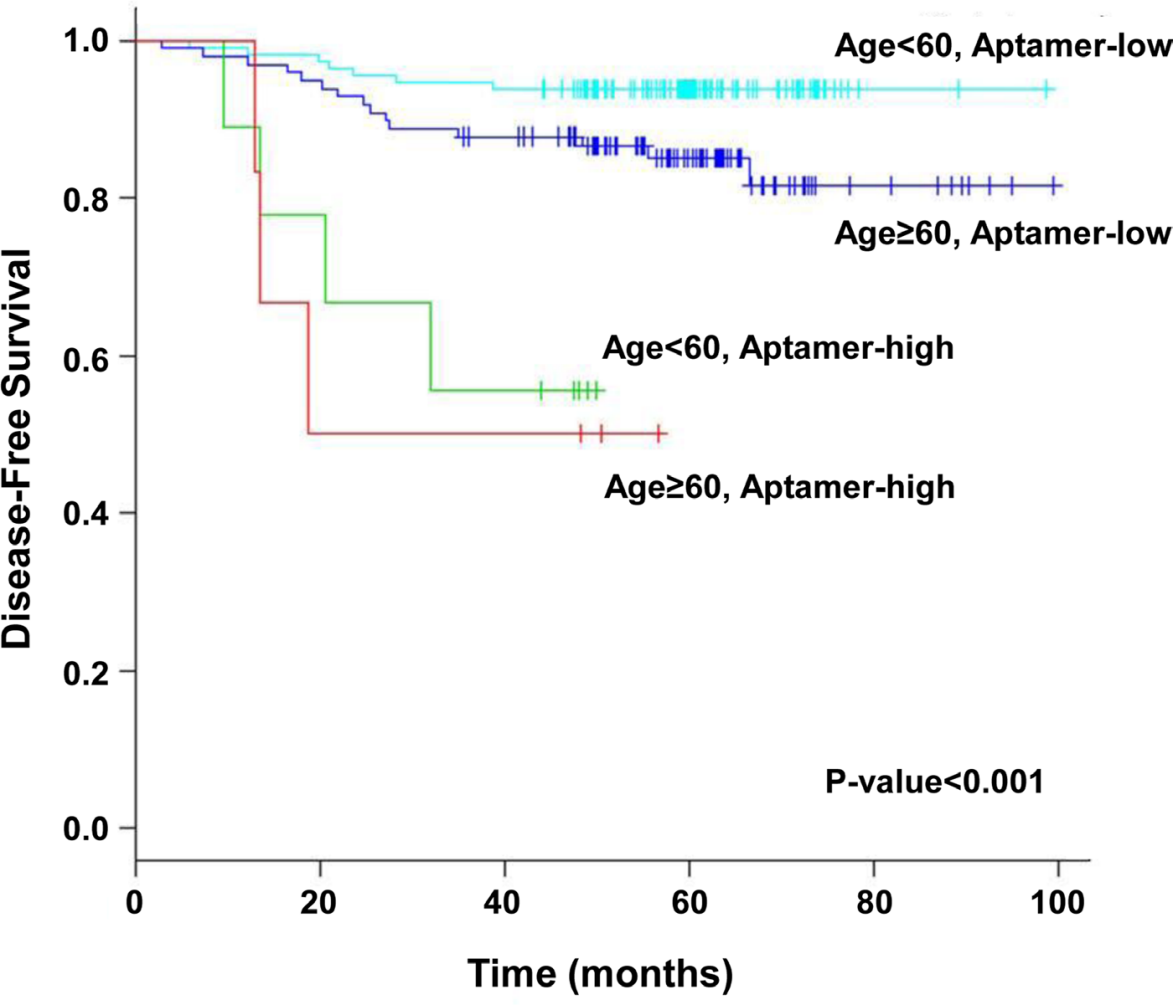
Analysis of DNA array using 1,149 aptamers Engineered RNA aptamers were prepared by *in vitro* transcription of DNA pool templates corresponding to 1,149 aptamers. An oligo array was prepared using a microarrayer with oligonucleotides generated from 1,149 aptamer sequences. Serum samples of colorectal cancer patients were allowed to react with nitrocellulose membrane in a tube. SELEX buffer and 1,149 aptamers were then added to the reaction tube. The nitrocellulose membrane was washed and heated at 95°C for 5 min, and the acquired solution was amplified using RT-PCR and labeled with Cy5. Equal amounts of Cy5-labeled and Cy3-labeled products from the above-mentioned RT-PCR of 1,149 aptamers were used as probes to react with the oligo array to generate result images, which were analyzed based on a constructed database with clinical information.

As shown in Figure 2, the single-stranded nucleic acids captured on the aptamer array were incubated at 60°C for 4 to 12 hours with the serum molecule-binding aptamers prepared in Example 3 to form pre-hybrids. The arrays were then washed at 42°C with 0.1X saline-sodium citrate (SSC) buffer. We used a hybridization solution containing 1 M NaCl, 0.3 M sodium citrate, 0.5% sodium dodecyl sulfate (SDS) or 100 μg/mL salmon sperm DNA, and 0.2% bovine serum albumin or single-stranded nucleic acids. After completion of the pre-hybridization, the glass slide was treated with the solution prepared in Example 3 at 42°C for 12 hours to conduct hybridization, followed by rinsing with washing solutions. To wash the chip, the following solutions were used in the sequence listed at 42°C for 30 min for each solution: 1X SSC plus 0.2% SDS, 1X SSC plus 0.2% SDS, 0.5X SSC plus 0.2% SDS, and 0.01X SSC plus 0.2% SDS.

### Identification and analysis of spots on the aptamer array

After completing the washes in Example 4, the glass slide was dried by centrifugation and scanned using a GenePix4000 laser scanner (Axon Instruments, Inc., Foster City, CA, USA). Laser light at a wavelength of 635 nm was used to excite the fluorescent dye (Cy5). Fluorescent images were captured as multiple-image-tagged image files and analyzed with GenePix Pro 3.0 software (Axon Instruments, Inc.).

### Prognostic model building and validation

We performed print-tip lowest normalization for each array and scale normalization. [5] Missing expression values were input using the k-nearest neighbor method with *k* = 10, and the arrays were standardized. [6] There were 1,156 aptamers and 227 samples included in statistical analysis. The gradient lasso algorithm was applied to fit a prediction model based on the Cox proportional hazards model and leave-one-out cross-validation (LOOCV) was used to evaluate the predictive performance. [7, 8] At each leave-one-out (LOO) step, the risk score was computed as the linear combination of expression values and their regression estimates developed from a training set (a set of size n-1) and the patient who was left out for testing was assigned to either the low or high risk group using a cut-off value for the risk scores. The optimal cut-off was chosen as the point with the most significant log-rank p-value for all possible cut-off points among n risk scores fitted through a LOO procedure. We calculated the log-rank test statistic value to compare the recurrence-free survival between the high and low risk groups from the LOO procedure applied to the original data set. To remove the overfitting bias of the log-rank p-value obtained from the LOO of the original data, a permutation p-value was calculated from 500 permutations. [9] From each permutation data set, we obtained an optimal cutoff value and a log-rank p-value, and the unbiased permutation p-value was estimated as the proportion of permutations whose log-rank p-value from the LOO procedure was smaller than that from the original data. Clinical risk was defined as poorly differentiated histology, lymphovascular invasion, and perineural invasion.

### Statistical analysis

Comparisons between the two groups for categorical variables were analyzed using the chi-square test or Fisher's exact test. Survival rates were estimated using the Kaplan-Meier method, and survival distributions between the two groups were compared using log-rank tests. A Cox proportional hazards model was used to determine which risk factors had an independent effect on survival after recurrence. P ≤ 0.05 was considered statistically significant. Statistical analyses were conducted using SPSS software, version 14.0 (SPSS Inc., Chicago, IL, USA) and R (version 2.12.0: www.r-project.org).
